# Comparison of the efficacy and safety of fruquintinib and regorafenib in the treatment of metastatic colorectal cancer: A real-world study

**DOI:** 10.3389/fonc.2023.1097911

**Published:** 2023-03-03

**Authors:** Ya-Ya Deng, Xin-Yue Zhang, Peng-Fei Zhu, Hong-Rui Lu, Qian Liu, Shuang-Yue Pan, Zhe-Ling Chen, Liu Yang

**Affiliations:** ^1^ Cancer Center, Department of Medical Oncology, Zhejiang Provincial People's Hospital (Affiliated People's Hospital, Hangzhou Medical College), Hangzhou, Zhejiang, China; ^2^ Graduate School of Clinical Medicine, The Qingdao University Medical College, Qingdao, Shandong, China; ^3^ Graduate School of Clinical Medicine, Bengbu Medical College, Bengbu, Anhui, China; ^4^ Graduate School of Clinical Medicine, Zhejiang Chinese Medical University, Hangzhou, Zhejiang, China

**Keywords:** fruquintinib, regorafenib, immunotherapy, advanced colorectal cancer, real-world study

## Abstract

**Background:**

Fruquintinib and regorafenib have been approved for the third-line therapy of metastatic colorectal cancer (mCRC) in China. However, at present, there is a lack of head-to-head clinical trials on the comparison of efficacy and safety between the two drugs.

**Materials and methods:**

The data of patients with mCRC who were treated with fruquintinib or regorafenib after the standard chemotherapy in Zhejiang Provincial People’s Hospital from October 2018 to November 2021 were collected and analyzed. The primary endpoints were overall survival (OS), progression-free survival (PFS) and adverse events. The secondary endpoints were the appropriate sequence, objective remission rate (ORR) and disease control rate (DCR) of fruquintinib and regorafenib.

**Results:**

A total of 105 patients were enrolled in this study. The ORR of fruquintinib group (n=55) and regorafenib group (n=50) were 6.1% and 2.0%; the DCR were 65.3% and 54.2%, respectively. There was no significant difference in median OS (mOS) and PFS (mPFS) between the two groups (mOS:14.2 vs12.0 months, p=0.057; mPFS:4.4 vs 3.5 months, p=0.150). Combined immunotherapy showed a synergistic effect. The mPFS and mOS of fruquintinib combined with anti-PD-1 therapy were longer than those of fruquintinib monotherapy (mPFS:5.9 vs 3.0 months, p=0.009; mOS:17.5 vs 11.3 months, p=0.008). The mOS of patients treated with regorafenib combined with anti-PD-1 therapy was 14.8 months higher than that of regorafenib monotherapy (p=0.045). When combined with anti-PD-1 therapy, the mPFS and mOS of fruquintinib was significantly longer than regorafenib (mPFS:5.9 vs 3.8 months, p=0.018; mOS:17.5 vs 14.8 months, p=0.044). In the treatment sequence, the OS of patients treated with regorafenib and then fruquintinib was significantly longer than that of the reverse treatment sequence (15.0 vs 8.3 months, p=0.019). The adverse reactions were generally similar, but the incidence of hand-foot syndrome of regorafenib was higher than that of fruquintinib, while fruquintinib was more prone to grade 3 hypertension.

**Conclusion:**

Fruquintinib monotherapy showed better disease control rate and objective remission rate in the post-line therapy of metastasis colorectal cancer. Notably, the combination of PD-1 immunotherapy brought the additional effect, especially in the fruquintinib combined with anti-PD-1 therapy. Patients treated with regorafenib and then fruquintinib was significantly longer than that of the reverse treatment sequence. The toxicity of fruquintinib and regorafenib are similar.

## Introduction

Colorectal cancer (CRC) is the third most common malignant tumor worldwide, and its mortality is second only to lung cancer (1). More than 20% of patients have metastasis at the time of initial diagnosis, most of them have unresectable tumors, and 25% of patients will develop metastasis after radical surgery (2). Standard treatment for these patients includes chemotherapy based on cytotoxic drugs (fluorouracil, oxaliplatin, irinotecan) and targeted therapy for vascular endothelial growth factor (VEGF; bevacizumab) and epidermal growth factor receptor (EGFR; cetuximab) (3). However, it is a pity that most patients with mCRC still have disease progression after first-or second-line standard treatment. At present, the choice of drugs for third-line or post-line treatment of mCRC is very limited.

Regorafenib is an oral multi-target kinase inhibitor, which inhibits tumorigenesis, tumor angiogenesis and tumor microenvironment by targeting VEGFR1/2/3, TIE-2, BRAF, KIT, RET, PDGFR and FGFR (4). The results of international randomized phase III CORRECT clinical trial show that regorafenib can prolong the survival time of chemotherapy-resistant patients with advanced CRC (5). The results of the subsequent phase III CONCUR clinical trial in Asia also show that the mOS and mPFS of patients with refractory mCRC were 8.8 months and 3.2 months respectively, significantly longer than those in the placebo group (6). Fruquintinib is a highly selective oral tyrosine kinase inhibitor targeting VEGFR1/2/3, which has a strong inhibitory effect on a variety of advanced tumors by inhibiting tumor angiogenesis (7, 8). The FRESCO study of phase III clinical study showed that the mOS in the fruquintinib group was significantly longer than that in the placebo group (9.3 vs 6.6 months, p<0.001), and the mPFS was significantly prolonged by 1.9 months (3.7 vs 1.8months, p<0.001) (9). Based on the above results, fruquintinib and regorafenib have been approved for third-line treatment of advanced CRC in China. However, there are still some questions about the application of these two multi-target kinase inhibitors.

Firstly, there is a lack of head-to-head clinical trials to confirm whether the efficacy and adverse reactions of the two drugs are different. A limited number of meta-analyses have indicated that the efficacy of fruquintinib and regorafenib is similar, but there is no consistent conclusion about the toxicity of the two drugs (10, 11). Second, as both drugs are approved and both are covered by health insurance in China, clinicians should prioritize which drug to use as a third-line treatment. Finally, the efficacy of fruquintinib or regorafenib alone is not significant. How to enhance the clinical efficacy of the two drugs or combine them with other treatment methods is still an urgent topic for clinical exploration. It is well known that tumors grow and evolve through continuous crosstalk with the surrounding microenvironment, and emerging evidence shows that angiogenesis and immunosuppression frequently occur simultaneously in response to this crosstalk. Accordingly, strategies combining anti-angiogenic therapy and immunotherapy seem to have the potential to tip the balance of the tumor microenvironment and improve treatment response (12).

Thus, we conducted this retrospective study to evaluate the efficacy and safety of fruquintinib and regorafenib in the real world and analyzed the rational use sequence of fruquintinib and regorafenib in patients with mCRC. Meanwhile, for the first time, we compared the continuing beneficial and adverse effects of long-term use of fruquintinib or regorafenib plus anti-PD-1 therapy.

## Patients and methods

### Patients

We collected information on patients with advanced CRC who were treated in Zhejiang Provincial people’s Hospital from October 2018 to November 2021 and conducted this real-world retrospective study. The follow-up period was from the first use of fruquintinib or regorafenib to death, loss of follow-up, or the end of the study. The main inclusion criteria of this study include: 1. mCRC confirmed by histopathology, computed tomography (CT) or magnetic resonance imaging (MRI). 2. Patients who had progressed or could not tolerate standard chemotherapy after second-line chemotherapy were treated with at least one cycle of fruquintinib or regorafenib on the third or posterior line. 3. The performance status of the eastern cooperative tumor group (ECOG-PS) was 0-2; 4. The age is between 18 and 78 years old. 5. At least one assessable lesion is included. The main exclusion criteria include: 1. Severe organ dysfunction; 2. Non-third-line or later-line use of regorafenib or fruquintinib. The deadline for data collection is November 1, 2021. This study was performed in line with the principles of the Declaration of Helsinki (as revised in Fortaleza, Brazil, in October 2013) and the international standard of good Clinical practice (GCP), and was approved by the Ethics Committee of Zhejiang Provincial people’s Hospital (approval No.: QT2022269).

### Therapy schedule

The patients in the fruquintinib group were treated with fruquintinib 3-5mg, and the patients in the regorafenib group were treated with 80-160mg once a day for 21 days, 28 days as a treatment cycle. The dosage of the medicine was adjusted based the side effects and the tolerance of patients. Some patients accepted the combination of PD-1 inhibitors with fruquintinib or regorafenib, including: sintilimab (200mg, Q3W), camrelizumab (200mg, Q3W), tislelizumab (200mg, Q3W), pembrolizumab (200mg, Q3W), nivolumab (240mg, Q3W), toripalimab (240mg, Q3W). Treatment will no longer continue in the event of disease progression, intolerable toxicity or other reasons that require discontinuation of treatment.

### Primary and secondary endpoints

The OS, PFS and adverse events (AEs) of fruquintinib and regorafenib were the main endpoints of this study, while the appropriate sequences, objective remission rate and disease control rate of fruquintinib and regorafenib were the secondary endpoints. The additional objective of this research is to compare the efficacy of fruquintinib and regorafenib combined immunotherapy.

### Assessment

Clinicians and researchers measured tumor lesions by CT or MRI every 2-3 treatment cycles, and evaluated tumor response according to The Response Evaluation Criteria in Solid Tumors (RECIST) Version 1.1. The observed drug-related adverse reactions were classified and graded according to The Common Terminology Criteria for Adverse Events (CTCAE) Version 5.0.

### Statistical analysis

SPSS version 25.0 (IBM Corp., Armonk, N.Y., USA) and GraphPad Prism 5.0 (GraphPad Software, San Diego, California, USA) were used for statistical analysis. The continuous variables in the baseline characteristics were compared by independent sample T-test, and the categorical variables in the baseline characteristics and treatment responses were assessed using χ2 test, Fisher exact test and Mann Whitney U nonparametric test. The survival curve was drawn by Kaplan-Meier method and Log-rank test was carried out. Cox proportional regression model was used to analyze the survival and prognosis. The significant factors (p<0.1) determined by univariate Cox regression analysis were included in multivariate Cox regression analysis, and the hazard ratio (HR) and confidence interval (CI) were calculated. The difference was statistically significant with p<0.05.

## Results

### Patient characteristics

**Figure 1:**
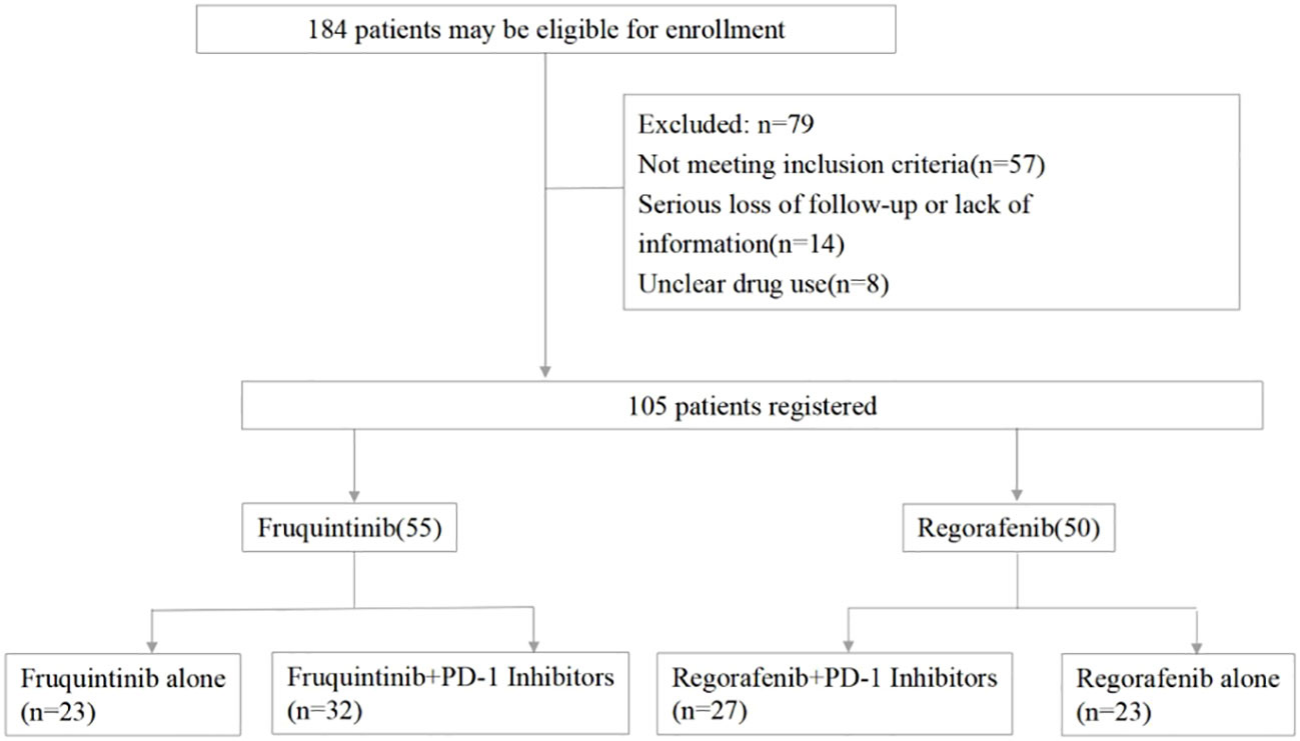
Flow diagram. PD-1, programmed death receptor-1.

184 patients were screened altogether, and only 105 patients met all the criteria and were enrolled in this study, including 55 patients in the fruquintinib group and 50 patients in the regorafenib group, and 32 patients in the fruquintinib group and 27 patients in the regorafenib group were treated with PD-1 inhibitors (**Figure 1**).

**Table 1** showed the baseline characteristics of all enrolled patients, including 80 (76.2%) males, 25 (23.8%) females and a median age of 63 years (32-78). Most of the patients (81.0%) were in good condition, with an ECOG scores of 0-1. 79 (75.2%) patients had left-colon tumor and 26 (24.8%) patients had right-colon tumor. The main distant metastases were liver metastasis (68.6%), lung metastasis (62.9%), peritoneal metastasis (27.6%) and bone metastasis (22.9%). Among the primary tumor gene mutations, 49 (46.7%) patients had a RAS mutation, 48 (45.7%) patients were RAS wild-type, 4 (3.8%) patients had a BRAF mutation and 93 (88.6%) patients were BRAF wild type. In the previous anti-tumor targeted therapy, 83.6% and 76.0% of the patients in the fruquintinib group and regorafenib group received anti-VEGF therapy, and 25.5% and 20.0% of the patients received anti-EGFR therapy. There was no statistical difference in baseline characteristics between the two groups.

**Table 1:** Baseline Characteristics.

Character	Total, n. (%)	Fruquintinib group, n. (%)	Regorafenib group, n. (%)	p-value
**Patients**	105	55	50	
**Sex**				0.965
Male	80 (76.2)	42 (76.4)	38 (76.0)	
Female	25 (23.8)	13 (23.6)	12 (24.0)	
**Age (years, median (range))**	62 (32-78)	61 (32-78)	63 (41-78)	0.246
**Age**				0.794
<65 years	56 (53.3)	30 (54.5)	26 (52.0)	
≥65 years	49 (46.7)	25 (45.5)	24 (48.0)	
**Baseline ECOG PS**				0.196
0	40 (38.1)	24 (43.6)	16 (32.0)	
1	45 (42.9)	19 (34.5)	26 (52.0)	
2	20 (19.0)	12 (21.8)	8 (16.0)	
**Primary tumor location at first diagnosis**				0.236
Center	79 (75.2)	44 (80.0)	35 (70.0)	
Right	26 (24.8)	11 (20.0)	15 (30.0)	
**Primary disease site at first diagnosis**				0.751
Colon	55 (52.4)	28 (50.9)	27 (54.0)	
Rectum	50 (47.6)	27 (49.1)	23 (46.0)	
Multiple metastases at study entry				
Lung	66 (62.9)	38 (69.1)	28 (56.0)	0.166
Liver	72 (68.6)	39 (70.9)	33 (66.0)	0.588
Peritoneum	29 (27.6)	11 (20.0)	18 (36.0)	0.067
Bone	24 (22.9)	14 (25.5)	10 (20.0)	0.506
**Number of metastases sites at treatment start**				0.111
<3	63 (60.0)	29 (52.7)	34 (68.0)	
≥3	42 (40.0)	26 (47.3)	16 (32.0)	
MSI status				0.963
pMMR	93 (88.6)	49 (89.1)	45 (90.0)	
dMMR	4 (3.8)	2 (3.6)	2 (4.0)	
Unknown	7 (6.7)	4 (7.3)	3 (6.0)	
**RAS mutation status**				0.872
RAS wild type	48 (45.7)	24 (43.6)	24 (48.0)	
RAS mutant	49 (46.7)	27 (49.1)	22 (44.0)	
Unknown	8 (7.6)	4 (7.3)	4 (8.0)	
**BRAF V600E status**				0.513
Wild-type	93 (88.6)	50 (90.9)	43 (86.0)	
Mutation	4 (3.8)	1 (1.8)	3 (6.0)	
Unknown	8 (7.6)	4 (7.3)	4 (8.0)	
**Prior antitumor treatment**				0.787
Radical surgery	70 (66.7)	38 (69.1)	32 (64.0)	
Non-radical surgery	5 (4.8)	2 (1.8)	3 (6.0)	
Chemotherapy or Radiation therapy	30 (28.6)	15 (27.3)	15 (30.0)	
**Prior chemotherapy with VEGF and EGFR inhibitors**				0.479
Neither	12 (11.4)	4 (7.3)	8 (16.0)	
VEGF inhibitor	84 (80.0)	46 (83.6)	38 (76.0)	
EGFR inhibitor	24 (22.9)	14 (25.5)	10 (20.0)	
Both	15 (14.3)	9 (16.4)	6 (12.0)	
**Combination of PD-1 inhibitors**				0.666
with	59 (56.2)	32 (58.2)	27 (54.0)	
without	46 (43.8)	23 (41.8)	23 (46.0)	

ECOG PS, Eastern Cooperative Oncology Group performance status; MSI, microsatellite instability, pMMR, proficient mismatch repair; dMMR, different Mismatch Repair; VEGF, vascular endothelial growth factor; EGFR, epidermal growth factor receptor; PD-1, programmed death receptor-1.

### Treatment

All individuals received fruquintinib or regorafenib as the third- or later-line therapy after the failure of standard chemotherapy. In the fruquintinib group, the initial dose of fruquintinib was 3mg (n=31), 4mg (n=14) and 5mg (n=10) (**Supplementary Figure 1A**), and the final dose was 2mg (n=2), 3mg (n=21), 4mg (n=19) and 5mg (n=13) (**Supplementary Figure 1B**). In the fruquintinib group, the initial dose was 80mg (n=31), 120mg (n=11) and 160mg (n=8) (**Supplementary Figure 1D**), and the adjusted final dose was 40mg (n=1), 80mg (n=29), 120mg (n=13) and 160mg (n=7) (**Supplementary Figure 1E**). In terms of combined anti-PD-1 treatment, fruquintinib combined with PD-1 inhibitors (FP) accounted for 58.2% (32/55), while regorafenib combined with PD-1 inhibitor (RP) accounted for 54.0% (27/50). The most commonly used PD-1 inhibitors in the two groups were sintilimab, camrelizumab and tislelizumab, accounting for 56.25%, 25.00%, 9.38% and 44.44%, 29.63%, 7.41% respectively (**Supplementary Figures 1C, F**).

### Clinical efficacy

By the end of last follow-up (September 1, 2022), a total of 105 patients, 97 patients had completed the efficacy evaluation, and 8 patients had not received PFS evaluation owing to serious adverse reactions or other disease progression, including 6 individuals in the fruquintinib group and 2 individuals in the regorafenib group. Finally, 81.1% of the patients in the fruquintinib group completed the PFS assessment, 67.3% (37/55) eventually died, and 32.7% (18/55) patients were still in follow-up, while 48 (96.0%) PFS events and 43 (86.0%) deaths were observed in the regorafenib group, and 7 (14.0%) patients were still followed up.

**Table 2** summarized the clinical efficacy of each group. The best response achieved in both groups was partial remission (PR). There were 3 patients in the fruquintinib group, while only one in the regorafenib group achieved PR, 29 (59.2%) patients in the fruquintinib group achieved stable disease (SD), 17 (34.7%) patients received progressive disease (PD), while 25 (52.1%) patients in the regorafenib group achieved SD, and 22 (45.8%) patients experienced PD. Therefore, the ORR of the fruquintinib group was 6.1% (3/49), and the DCR was 65.3% (32/49), and the ORR and DCR of regorafenib group were 2.0% and 54.2%, respectively. Although the proportion of patients with ORR and DCR in fruquintinib group was higher than that in regorafenib group, the difference was not statistically significant.

**Table 2:** Curative effect evaluation.

Clinical efficacy	Fruquintinib group (49), n. (%)	Regorafenib group (49), n. (%)	p-value
Overall response			0.231
Complete response	0	0	
Partial response	3 (6.1)	1 (2.0)	
Stable disease	29 (59.2)	25 (52.1)	
Progressive disease	17 (34.7)	22 (45.8)	
Objective response rate	3 (6.1)	1 (2.0)	0.610
Disease control rate	32 (65.3)	26 (54.2)	0.218

In the subgroup analysis of fruquintinib and regorafenib monotherapy or combined immunotherapy, the DCR of fruquintinib monotherapy and regorafenib monotherapy were 54.5% and 47.6%, respectively, while the DCR and ORR of FP group and RP group were 74.1%, 7.4% and 59.3%, 3.7%, respectively. Although the DCR and ORR of fruquintinib were higher than those of regorafenib, whether alone or in combination with PD-1 inhibitor, no statistical significance was observed (**Supplementary Table 1**).

### Survival analysis

**Figure 2:**
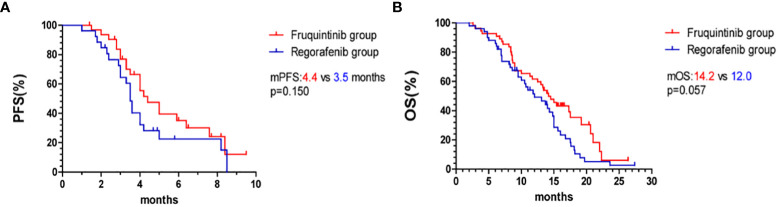
Kaplan–Meier curves. **(A)** PFS in the fruquintinib group and regorafenib group. **(B)** OS in the fruquintinib group and regorafenib group. mPFS, median progression-free survival. mOS, overall survival.

**Figure 3:**
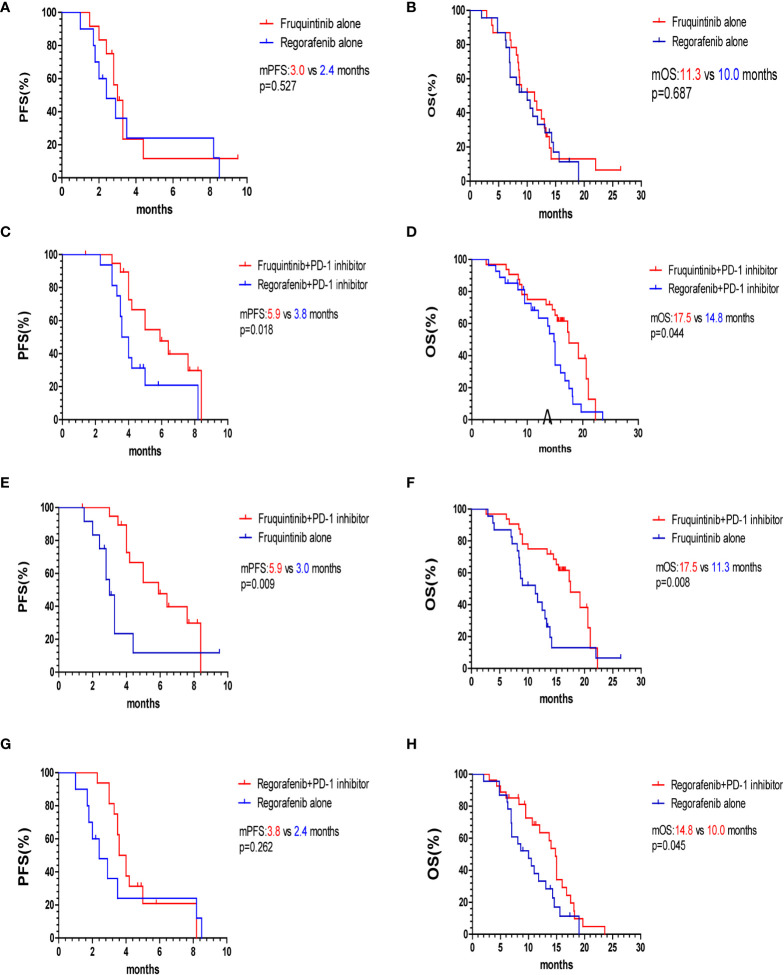
Kaplan–Meier survival curves of PFS and OS of two subgroups. **(A, B)** PFS and OS of fruquintinib and regorafenib monotherapy. **(C, D)** PFS and OS of FP and RP. **(E, F)** PFS and OS of fruquintinib with or without PD-1 inhibitors. **(G, H)** PFS and OS of regorafenib with or without PD-1 inhibitors. mPFS, median progression-free survival; mOS, overall survival; PD-1, programmed death receptor-1.

The overall survival curve is shown in **Figures 2**, **3**. The mPFS and mOS in fruquintinib group were 4.4 months and 14.2 months, and that of regorafenib group were 3.5 months and 12.0 months. There were no significant difference (p=0.150; p=0.057 **Figures 2A, B**). In the subgroup analysis of fruquintinib or regorafenib monotherapy, the median PFS of fruquintinib monotherapy and regorafenib monotherapy were 3.0 months and 2.4 months, respectively (p=0.527 **Figure 3A**), and the mOS was 11.3 months and 10 months respectively, and the difference was not statistically significant (p=0.687 **Figure 3B**). Subsequently, in the subgroup analysis of FP and RP, it was found that the median PFS of FP was significantly longer than that of RP (5.9 vs 3.8 months, p=0.018 **Figure 3C**). The median OS of FP and RP was 17.5 months and 14.8 months, respectively. Compared with the RP group, FP group has a longer OS (p=0.044 **Figure 3D**).

To further evaluate the survival benefits of immunotherapy, we compared the efficacy of fruquintinib or regorafenib monotherapy with that of combination with PD-1 inhibitors. The median PFS and OS of the FP group were significantly longer than that of the fruquintinib monotherapy group (p=0.009; p=0.008 **Figures 3E, F**). Compared with the regorafenib monotherapy group, the median OS of the RP group was prolonged (14.8 vs 10.0 months, p=0.045 **Figure 3H**). Although the PFS in the RP group showed an increasing trend compared with that in the regorafenib monotherapy group, there was no statistical significance (3.8 vs 2.4 months, p=0.262 **Figure 3G**).

### Treatment sequence

**Figure 4:**
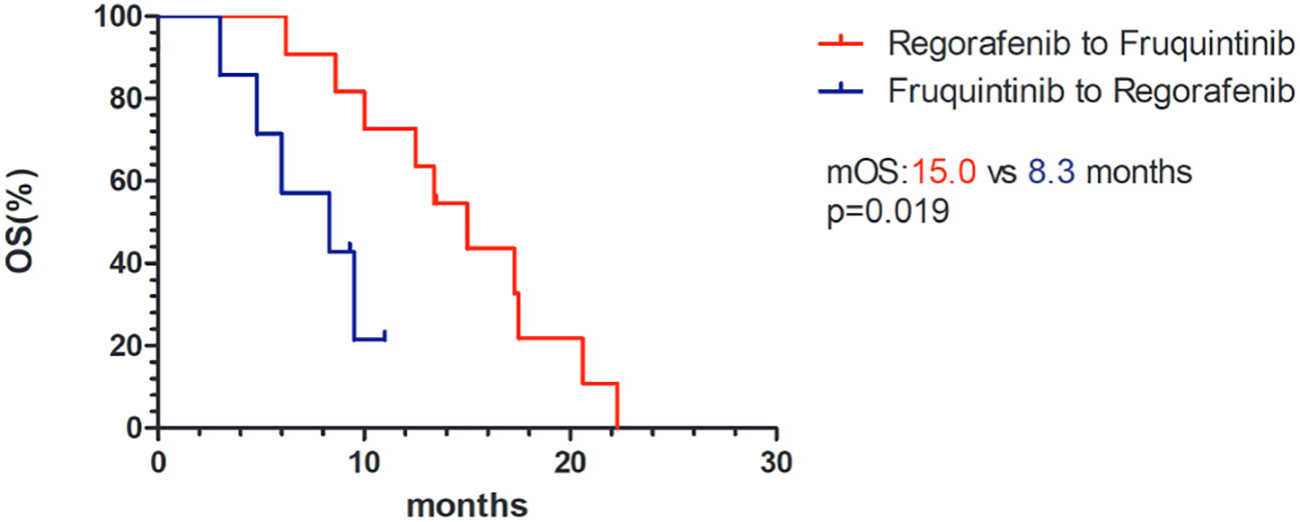
Kaplan–Meier survival curves of OS of different therapeutic sequences of fruquintinib and regorafenib. mOS, median overall survival.

In the fruquintinib group, 11 patients received fruquintinib after the progression of treatment with regorafenib, of which 8 (72.7%) achieved SD, while in the regorafenib group, 8 patients were treated with regorafenib after the progression of fruquintinib, of which only 3 (37.5%) controlled the progression of the disease, and the difference between the two was not statistically significant (p=0.181). However, the median OS of patients treated with fruquintinib after the progression of regorafenib treatment was significantly longer than that of the opposite treatment sequence (15.0 vs 8.3 months, p=0.019 **Figure 4**).

### Safety

The adverse reactions (AEs) of fruquintinib and regorafenib are shown in **Table 3**. In general, 43 (78.2%) of the 55 suffers in the fruquintinib group experienced at least one AE, while 44 (88.0%) suffers in the regorafenib group experienced at least one AE, and there was no significant difference between the two groups (χ2 = 1.78, p=0.182). 16 (29.1%) patients had at least one grade 3 adverse event in the fruquintinib group, of which 14 (25.5%) had treatment-related withdrawal or reduction. 11 (22.0%) patients in the regorafenib group had at least one grade 3 adverse event, of which 8 (16%) were stopped or reduced owing to intolerable drug toxicity. There were no deaths due to AEs. The most common AEs in the fruquintinib group were hypertension (34.5%), hand-foot syndrome (21.8%) and proteinuria (18.2%), while in the regorafenib group, hand-foot syndrome (44.0%), abnormal liver function (30.0%) and hypertension (20.0%) were the most common AEs. The incidence of hand-foot syndrome in the regorafenib group was higher than that in the fruquintinib group (χ2 = 5.89, p=0.015), while the probability of hypertensive events with ≥ 3 grade in the fruquintinib group was higher than that in the regorafenib group (χ2 = 4.22, p=0.040). It is worth mentioning that in the fruquintinib group, we observed for the first time the occurrence of aortic dissection associated with fruquintinib.

**Table 3:** Adverse reaction.

Adverse reaction	All			Grade 3-5		
	Fruquintinib group (55), n. (%)	Regorafenib group (50), n. (%)	p-value	Fruquintinib group (55), n. (%)	Regorafenib group (50), n. (%)	p-value
ALL	43 (78.2%)	44 (88.0%)	0.182	16 (29.1)	11 (22.0)	0.406
Hand–foot skin reaction	12 (21.8)	22 (44.0)	**0.015**	4 (7.3)	6 (12.0)	0.623
Hypertension	19 (34.5)	10 (20.0)	0.096	14 (25.5)	5 (10.0)	**0.040**
Proteinuria	10 (18.2)	4 (8.0)	0.125	3 (5.5)	0	0.140
Thrombocytopenia	5 (9.1)	3 (6.0)	0.820	1 (1.8)	1 (2.0)	1.000
Liver dysfunction (ALT, AST level elevated)	8 (14.5)	15 (30.0)	0.056	0	2 (4.0)	0.224
Bilirubin level elevated	7 (12.7)	8 (16.0)	0.632	0	0	NA
Fatigue	5 (9.1)	6 (12.0)	0.627	0	0	NA
Hoarseness	6 (10.9)	5 (10.0)	0.879	2 (3.6)	0	0.272
Oral mucositis	9 (16.4)	7 (14.0)	0.736	0	1 (2.0)	0.476
Decreased appetite	4 (7.3)	5 (10.0)	0.881	1 (1.8)	0	0.524
Digestive tract reaction (Nausea/Vomiting)	2 (3.6)	3 (6.0)	0.913	1 (1.8)	0	0.524
Occult blood positive	5 (9.1)	6 (12.0)	0.627	1 (1.8)	0	0.524
Nose bleed	3 (5.5)	2 (4.0)	1.000	0	1 (2.0)	0.476
Diarrhoea	4 (7.3)	6 (12.0)	0.623	0	0	NA
Myodynia/Arthrodynia	6 (10.9)	1 (2.0)	0.151	0	0	NA
Aortic dissection	1 (1.8)	0	0.524	1 (1.8)	0	0.524
Acute kidney injury	1 (1.8)	0	0.524	1 (1.8)	0	0.524

NA, no available; The bold values represent P<0.05, and the difference is statistically significant.

### Analysis of prognostic factors of combined immunotherapy with fruquintinib or regorafenib

In the univariate analysis of 59 patients treated with combined immunotherapy, ECOG-PS score and fruquintinib or regorafenib targeted therapy were significantly correlated with PFS. Previous anti-VEGF therapy and anti-EGFR therapy were factors with p<0.1. In multivariate analysis, ECOG-PS score, previous anti-VEGF therapy and fruquintinib or regorafenib targeted therapy were significant independent predictors of PFS (**Table 4**). In the univariate analysis of OS, ECOG-PS score, liver metastasis and previous antineoplastic therapy were associated with OS. RAS mutation, fruquintinib or regorafenib targeted therapy and previous anti-VEGF therapy were factors with p<0.1. In multivariate analysis, ECOG-PS score and fruquintinib or regorafenib targeted therapy were independent predictors of OS (**Table 5**).

**Table 4:** Univariate and multivariate analyses of risk factors for PFS of FP and RP.

	Univariate analysis			Multivariate analysis		
	HR	95%CI	p-value	HR	95%CI	p-value
Age						
≥65 vs <65	1.05	0.47-2.36	0.905			
Sex						
female vs male	1.19	0.44-3.22	0.726			
ECOG PS						
0 vs 1 vs 2	**2.37**	**1.21-4.66**	**0.012**	**2.18**	**1.05-4.54**	**0.037**
Tumor location						
left vs right	1.43	0.53-3.85	0.478			
Site of primary tumor						
rectum vs colon	0.83	0.37-1.87	0.649			
Liver metastasis						
yes vs no	2.00	0.85-4.69	0.112			
Lung metastases						
yes vs no	1.11	0.47-2.63	0.815			
Peritoneal metastases						
yes vs no	1.42	0.51-3.97	0.500			
Number of distant metastatic sites						
≥3 vs <3	1.48	0.66-3.31	0.343			
KRAS or NRAS mutation						
yes vs no	1.61	0.63-4.08	0.318			
BRAF mutation						
yes vs no	0.73	0.10-5.51	0.762			
Prior antitumor treatment						
surgery vs chemotherapy or radiation therapy	0.66	0.26-1.67	0.379			
Targeted drugs						
fruquintinib vs regorafenib	**2.52**	**1.10-5.76**	**0.028**	**3.05**	**1.26-7.38**	**0.014**
Previous anti-VEGF treatment						
yes vs no	**2.72**	**0.89-8.34**	**0.080**	**3.62**	**1.17-11.23**	**0.026**
Previous anti-EGFR treatment						
yes vs no	**2.40**	**1.00-5.77**	**0.051**	2.12	0.88-5.08	0.093

ECOG PS, Eastern Cooperative Oncology Group performance status; VEGF, vascular endothelial growth factor; EGFR, epidermal growth factor receptor. The bold value in the univariate analysis represents p<0.1 and is included in the multivariate analysis; The bold values in multivariate analysis represent p<0.05, and the difference is statistically significant.

**Table 5:** Univariate and multivariate analyses of risk factors for OS of FP and RP.

	Univariate analysis			Multivariate analysis		
	HR	95%CI	p-value	HR	95%CI	p-value
Age						
≥65 vs <65	0.59	0.31-1.14	0.114			
Sex						
female vs male	0.91	0.41-2.01	0.811			
ECOG PS						
0 vs 1 vs 2	**2.42**	**1.41-4.17**	**0.001**	**2.80**	**1.35-5.80**	**0.006**
Tumor location						
Left vs right	1.02	0.47-2.25	0.956			
Site of primary tumor						
Rectum vs colon	1.09	0.58-2.06	0.779			
Liver metastasis						
yes vs no	**2.35**	**1.16-4.77**	**0.018**	1.48	0.62-3.52	0.374
Lung metastases						
yes vs no	0.69	0.34-1.38	0.293			
Peritoneal metastases						
yes vs no	0.95	0.45-2.02	0.901			
Number of distant metastatic sites						
≥3 vs <3	0.89	0.46-1.73	0.727			
KRAS or NRAS mutation						
yes vs no	**1.91**	**0.98-3.73**	**0.059**	1.59	0.73-3.46	0.244
BRAF mutation						
yes vs no	0.84	0.11-6.29	0.863			
Prior antitumor treatment						
surgery vs chemotherapy or radiation therapy	**0.40**	**0.20-0.82**	**0.012**	0.58	0.25-1.32	0.19
Targeted drugs						
fruquintinib vs regorafenib	**1.89**	**1.00-3.56**	**0.050**	**2.53**	**1.21-5.27**	**0.014**
Previous anti-VEGF treatment						
yes vs no	**2.39**	**0.90-6.33**	**0.080**	1.06	0.33-3.35	0.928
Previous anti-EGFR treatment						
yes vs no	1.12	0.56-2.24	0.741			

ECOG PS, Eastern Cooperative Oncology Group performance status; VEGF, vascular endothelial growth factor; EGFR, epidermal growth factor receptor. The bold value in the univariate analysis represents p<0.1 and is included in the multivariate analysis; The bold values in multivariate analysis represent p<0.05, and the difference is statistically significant.

## Discussion

As the choice of third-line therapy for mCRC, there are no clinical trials comparing the efficacy and safety of fruquintinib and regorafenib. Our research showed that fruquintinib and regorafenib have similar median overall survival (14.2 versus 12.0 months) and median progression-free survival (4.4 versus 3.5 months). Compared with FRESCO study and CONCUR study, our results are superior to these two key clinical studies, mainly due to the fact that in the real world, in addition to receiving fruquintinib or regorafenib, in order to control the progression of the disease, patients usually receive immunotherapy, local therapy or other forms of combination therapy, thus improving their survival prognosis.

Subsequently, we analyzed the different treatment sequence of fruquintinib and regorafenib, and the median OS of fruquintinib after the progression of treatment with regorafenib was significantly longer than that of the reverse treatment order. In terms of PFS, although the difference was not statistically significant, it may be related to the small sample size of this study. The results of the REVERCE study showed that the OS of mCRC patients who received sequential cetuximab treatment with regorafenib after completion of 6 cycles of chemotherapy was significantly longer than that of patients treated in the opposite order (mOS:17.4 vs 11.6 months, HR=0.61, p=0.029) (13). The work of Eraslan et al. has revealed that re-chemotherapy after the failure of regorafenib treatment can still prolong the survival time of patients with mCRC (14). These results showed that the efficacy of other treatments was cited after the progress of regorafenib treatment. Nevertheless, the mechanism of fruquintinib is different from that of cetuximab and chemotherapy. We surmise that: on the one hand, fruquintinib can maintain higher drug exposure. Due to the inhibition of multiple targets, the drug exposure at the maximum tolerance dose (MTD) is limited, resulting in poor inhibition and/or short duration of any target (especially VEGFR). Fruquintinib has high selectivity to VEGFR1/2/3, which can minimize miss toxicity and provide higher dose of drug exposure under MDT. On the other hand, fruquintinib had almost the same inhibitory effect on VEGFR2 and VEGFR3. VEGF-A/VEGFR1 mainly regulates angiogenesis, while VEGF-C/VEGFR3 regulates both vascular and lymphatic angiogenesis, promoting lymphatic vessel-mediated regional lymph node metastasis (15). Fruquintinib has similar activity to VEGFR2 and VEGFR3 and has higher selectivity than regorafenib, which can simultaneously block the blood vessels and lymphatic vessels affecting tumor growth and metastasis (16). Compared with Regorafenib, which mainly targets VEGF-A/VEGFR2, it can further inhibit tumor progression.

In the subgroup analysis of fruquintinib and regorafenib monotherapy or combined immunotherapy, the mPFS, ORR and DCR of fruquintinib and regorafenib monotherapy were 3.0 months, 4.5%, 54.5% and 2.4 months, 0, 47.6%, respectively. The results of the study were lower than those of the FRESCO study or CONCUR study. This may be related to the fact that the overall condition of the patients included in the clinical trial is good and that the dose of fruquintinib or regorafenib in most patients in this study did not reach the standard dose of the clinical trial (fruquintinib 5mg/d, regorafenib 160mg/d). Studies have shown that, first of all, antiangiogenic small molecule tyrosine kinase inhibitor can target VEGFR, normalize tumor vessels, thus improve tumor cell hypoxia and promote effective T cell infiltration into tumor tissue (17). Secondly, antiangiogenic drugs can inhibit the production of Tregs, TAM and MDSC in tumor sites and down-regulate the expression of immunosuppressive factors, which leads to the reprogramming of immunosuppressive microenvironment to immunostimulatory microenvironment (18, 19). Finally, antiangiogenic drugs can enhance the efficacy of immunotherapy by promoting antigen presentation and activating cytotoxic CD8+T cells (20). The crosstalk between angiogenesis and immune cells provides a theoretical basis for immunotherapy combined with antiangiogenic targeted therapy. In our study, the mOS and mPFS of FP were 17.5months and 5.9months, respectively. Compared with fruquintinib monotherapy, immunotherapy played a synergistic effect. At present, the data on the efficacy of fruquintinib combined with immunotherapy in real-world practice is limited. Phase I clinical trials show that the mPFS of fruquintinib combined with immunotherapy is 5.45-6.8 months (21, 22), which is similar to our results. In the RP group, the mOS was 14.8 months, and the mPFS was 3.8 months. The results of the Ib phase REGONIVO study conducted in Japan showed that the mPFS and ORR were 6.3 months and 40%, respectively (23). Our study did not achieve the surprising results of the REGONIVO study. This is related to the different baseline characteristics of the patients. In the phase Ib/II REGOTORI study conducted in China, the baseline characteristics of the patients included were similar to our research. 39 patients who received regorafenib combined with toripalimab, with a median PFS of 2.6 months, a median OS of 15.5 months, and an ORR of 15.2% (24). Another multicenter retrospective study of regorafenib combined with immune checkpoint inhibitors included 84 mCRC patients, with an ORR of 5% and a median PFS of 3.1 months (25). Obviously, our results are similar to those of the appeal study in China. Compared with the regorafenib monotherapy, the median PFS of the combination immunotherapy was longer, but the difference was not statistically significant, which may be related to the small number of patients enrolled in this study. The results of the multi-center retrospective study conducted by xu et al (26) revealed that compared with the monotherapy group, the combination immunotherapy of regorafenib could prolong the median PFS (3.5 vs 2.2 months, p=0.043). In the comparative analysis of FP and RP, the PFS and OS of the FP group were significantly better than those of the RP group. On the one hand, fruquintinib is a highly selective tyrosinase inhibitor, which has a stronger inhibitory effect on VEGFR1/2/3, while regorafenib is a multi-target inhibitor, which has less inhibitory effect on VEGF-VEGFR pathway than fruquintinib, which may also mean that the immunosuppressive effect of regorafenib in reversing VEGF is not as good as that of fruquintinib. On the other hand, the tolerance of fruquintinib is better than that of regorafenib. In our study, most patients took regorafenib from a low dose and then, based on the results of the ReDOS study (27), increased the dose weekly to the standard dose of 160mg/d. Fruquintinib was used to increase the dose up to 5mg/d based on a similar principle. Although the adverse reactions of fruquintinib and regorafenib were similar, fewer patients in the fruquintinib group had adverse reactions and more patients achieved dose increments.

With the wide application of immunotherapy in clinic, it is particularly important to select the dominant population of immunotherapy. In this study, the baseline characteristics of patients in the FP and RP group were analyzed for efficacy predictors. The results showed that ECOG-PS score, fruquintinib or regorafenib targeted therapy and previous anti-VEGF therapy were independent prognostic factors affecting PFS, and patients who did not receive anti-VEGF therapy in previous anti-tumor therapy may have longer PFS than those who received anti-VEGF therapy. In the FRESCO study, a subgroup analysis of previous anti-VEGF or anti-EGFR targeted therapy found that the mOS and mPFS of patients who had not received anti-VEGF therapy were significantly longer than those who had received anti-VEGF (9). Another retrospective study also showed that the mPFS of patients previously treated with anti-VEGFR drugs was shorter than that of patients without anti-VEGFR drugs (1.9 vs 3.7 month, p=0.006), but there was no significant difference in mOS between the two groups (9.0 vs 8.5 months, p=0.992) (28). ECOG-PS score, targeted therapy with fruquintinib or regorafenib were independent predictors of OS in patients. It should be pointed out that the physical status (PS) score of patients not only affects the total survival time of patients, but also affects the progression-free survival time of patients with drug treatment, and the patients with higher PS score are related to poor prognosis.

With regard to the adverse events (AEs) of the two drugs, meta-analysis of 1380 patients included by Jing et al. showed that fruquintinib was less toxic than regorafenib (10). However, Chen et al. included a meta-analysis of 2604 mCRC patients in five randomized controlled clinical trials that found that fruquintinib significantly increased the risk of serious adverse events (SAEs) compared with regorafenib (11). In the present study, although the toxicity of the two drugs was generally similar, the proportion of patients taking fruquintinib had fewer AEs than that of regorafenib, and more patients increased the dose. It seemed that fruquintinib was better tolerated than regorafenib. Nevertheless, SAEs accounted for 27.1% (29/107) of any grade of AEs in the fruquintinib group, while only 18% (16/89) in the regorafenib group. It seems that fruquintinib would increase the risk of SAEs, which may be related to the high selectivity of fruquintinib to the target.

In this study, without directly comparing the randomized controlled trials of regorafenib and fruquintinib, we evaluated the similar efficacy and safety of regorafenib and fruquintinib in the real world, explored the rational use sequence of regorafenib followed by fruquintinib and summarized the relevant molecular mechanisms. Meanwhile, we also compared for the first time the degree of sustained beneficial and adverse effects of long-term administration of fruquintinib or regorafenib combined with anti-PD-1 treatment. However, this research still has some limitations. Firstly, this study is a single-center retrospective study with a small sample size and a certain selection deviation. Prospective randomized clinical trials are still needed to verify our results in the future. Secondly, although the type and proportion of PD-1 inhibitors used in combination with fruquintinib or regorafenib are similar in this study, it will inevitably affect the consistency of the treatment process. Third, MSS status is not available in a small number of patients, but only a small number of patients with MSI-H, this restriction may not cause much deviation. Fourth, the PD-L1 CPS and TMB status of the included patients is unknown, resulting in the inability to determine the advantage of immunotherapy. Finally, this study is a real-world study, and not all patients have been tested for RAS and BRAF genes, which may limit the analysis of the efficacy of drug therapy.

## Conclusion

In summary, the efficacy and toxicity of fruquintinib and regorafenib in the treatment of advanced CRC are similar, but the incidence of hand-foot syndrome of regorafenib is higher than that of regorafenib, and fruquintinib is more prone to grade 3 hypertension. Fruquintinib can still prolong the survival time of patients after the progression of regorafenib treatment. Combined immunotherapy has a synergistic effect, which is more obvious in fruquintinib.

## Data availability statement

The original contributions presented in the study are included in the article/**Supplementary Material**. Further inquiries can be directed to the corresponding authors.

## Ethics statement

The studies involving human participants were reviewed and approved by The Ethics Committee of Zhejiang Provincial people’s Hospital. The ethics committee waived the requirement of written informed consent for participation. Written informed consent was obtained from the individual(s) for the publication of any potentially identifiable images or data included in this article.

## Author contributions

LY and Z-LC made contribution to conception and design. Y-YD analyzed and interpreted the data. Y-YD, X-YZ, P-FZ, H-RL, QL, and S-YP performed the data collection. Y-YD and XZ prepared the final draft. All authors contributed to the article and approved the submitted version.

## Supplementary material

Supplementary Figure 1 Initial and final doses of fruquintinib and regorafenib and types of combined PD-1 inhibitors. **(A, B)** Initial and final dose of fruquintinib. **(C)** Types and proportion of fruquintinib combined with PD-1 inhibitors. **(D, E)** Initial and final dose of regorafenib. **(F)** Types and proportion of regorafenib combined with PD-1 inhibitors.
