# Supplementary material for: Comparison of the efficacy and safety of fruquintinib and regorafenib in the treatment of metastatic colorectal cancer: A real-world study
Source: Front Oncol. 2023 Mar 3;13:1097911. doi: 10.3389/fonc.2023.1097911 (PMC10020225; doi:10.3389/fonc.2023.1097911)
Supplement: Supplementary file 1 [file Table_1.doc]

***Supplementary Table 1*** Clinical efficacy of fruquintinib or regorafenib in monotherapy or combined immunotherapy.

| Clinical efficacy | Fruquintinib alone (n=22),  n. (%) | Regorafenib alone (n=21), n. (%) | p-value | FP (n=27),  n. (%) | RP (n=27), n. (%) | p-value |
| --- | --- | --- | --- | --- | --- | --- |
| Overall response, n. (%) |  |  | 0.665 |  |  | 0.281 |
| Complete response | 0 | 0 |  | 0 | 0 |  |
| Partial response | 1(4.5) | 0 |  | 2(7.4) | 1(3.7) |  |
| Stable disease | 11(50) | 10(47.6) |  | 18(66.7) | 15(55.6) |  |
| Progressive disease | 10(45.5) | 11(52.4) |  | 7(25.9) | 11(40.7) |  |
| Objective response rate, n. (%) | 1(4.5) | 0(0) | 1.000 | 2(7.4) | 1(3.7) | 1.000 |
| Disease control rate, n. (%) | 12(54.5) | 10(47.6) | 0.650 | 20(74.1) | 16(59.3) | 0.248 |
