# Supplementary figures and images for: Comparison of the efficacy and safety of fruquintinib and regorafenib in the treatment of metastatic colorectal cancer: A real-world study
Source: Front Oncol. 2023 Mar 3;13:1097911. doi: 10.3389/fonc.2023.1097911 (PMC10020225; doi:10.3389/fonc.2023.1097911)

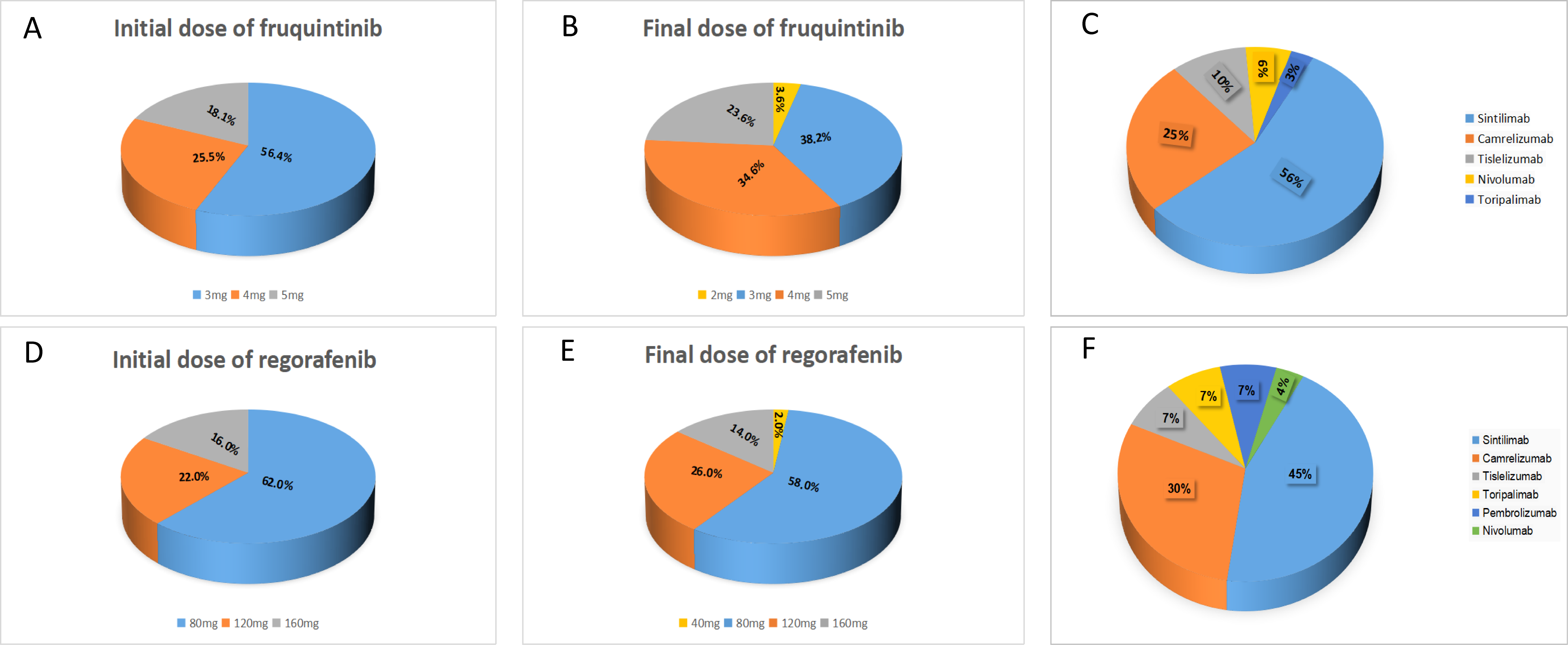

Supplement: Supplementary Figure 1 — Initial and final doses of fruquintinib and regorafenib and types of combined PD-1 inhibitors. (A, B) Initial and final dose of fruquintinib. (C) Types and proportion of fruquintinib combined with PD-1 inhibitors. (D, E) Initial and final dose of regorafenib. (F) Types and proportion of regorafenib combined with PD-1 inhibitors. [file Image_1.tif]
